# Clinical reminder alert fatigue in healthcare: a systematic literature review protocol using qualitative evidence

**DOI:** 10.1186/s13643-017-0627-z

**Published:** 2017-12-13

**Authors:** Ruth Backman, Susan Bayliss, David Moore, Ian Litchfield

**Affiliations:** Institute of Applied Health Research, University of Birmingham, Edgbaston, Birmingham, B15 2TT UK

**Keywords:** Computerised clinical decision support systems, Alerts, Clinical reminders, Alert fatigue, Clinical reminder fatigue, Systematic review, Protocol, Qualitative

## Abstract

**Background:**

Integrated reminders within clinical systems have become more prevalent due to the use of electronic health records and evidence demonstrating an increase in compliance within practice. Clinical reminders are assessed for effectiveness on an individual basis, rather than in combination with existing prompts for other conditions. The growing number of prompts may be counter-productive as healthcare professionals are increasingly suffering from “reminder fatigue” meaning many reminders are ignored. This work will review the qualitative evidence to identify barriers and enablers of existing prompts found within computerised decision support systems. Our focus will be on primary care where clinicians have to negotiate a plethora of reminders as they deal with increasingly complex patients and sophisticated treatment regimes. The review will provide a greater understanding of existing systems and the way clinicians interact with them to inform the development of more effective and targeted clinical reminders.

**Methods:**

A comprehensive search using piloted terms will be used to identify relevant literature from 1960 (or commencement of database) to 2017. MEDLINE, MEDLINE In Process, EMBASE, HMIC, PsycINFO, CDSR DARE, HTA, CINAHL and CPCI, will be searched, as well as grey literature and references and citations of included papers. Manuscripts will be assessed for eligibility, bias and quality using the CASP tool with narrative data being included and questionnaire based studies excluded. Inductive thematic analysis will be performed in order to produce a conceptual framework defining the key barriers around integrated clinical reminders.

**Discussion:**

Indications of alert and reminder fatigue are found throughout the current literature. However, this has not been fully investigated using a robust qualitative approach, particularly in a rapidly growing body of evidence. This review will aid people forming new clinical systems so that alerts can be incorporated appropriately.

**Systematic review registration:**

PROSPERO: CRD42016029418

**Electronic supplementary material:**

The online version of this article (10.1186/s13643-017-0627-z) contains supplementary material, which is available to authorized users.

## Background

Implementation of desirable clinical behaviours, such as compliance with national guidelines, has taken a variety of forms within healthcare [1, 2]. One popular intervention is the use of an integrated electronic reminder or prompt [3], often taking the form of a ‘pop-up box’ within the clinical health record system [4, 5]. This type of prompt, usually delivered at the point of care, has two broad functions; reminding the user to perform a task, or to alert them about the potential consequences of not performing a task [6].

Approximately 90% of all patient consultations in the UK occur in primary care, equating to some 360 million appointments per year [7, 8]. The treatment of the majority of patients relies on guidelines designed to treat single diseases [9]. However, current estimates indicate that 2.9 million people in the UK will be diagnosed as having two or more chronic conditions by 2018 [10], leading to a rise in the number of reminders for each patient, particularly where treatments are combined [11, 12]. These clinical reminders are used to influence clinician’s behaviour in a number of aspects of care including prescribing [12]. In exploring the benefits of clinical support systems, a number of reviews have reported broadly positive effects. However, frequently these alerts are evaluated individually rather than in a cumulative fashion; furthermore, there has been a dramatic rise in the number of prompts, or reminders. Many are integrated within electronic health records and should be activated at the point of care to promote shared decision-making [13–15]. However, the numbers of prompts combined with increasing time pressures in the National Health Service (NHS) is increasing the cognitive load within this working environment [16], to such an extent that these reminders are largely being disabled, thus negating their effectiveness [17]. This failure to engage with these digital prompts has been termed ‘alert fatigue’. By manually disabling all prompts, regardless of content, there are implications for patient safety, such as those providing warnings over medication [18], but also the increased interruption to the patient consultation can lead to a potential increase in medicolegal risk [19].

The aim of this research is to identify the barriers and enablers in the use of alerts and reminders within clinical systems as reported by healthcare professionals. Our focus will be within primary care and the electronic alerts found in this setting, including but not limited to alerts for prescribing, practice performance and lifestyle advice. This will be achieved by conducting a systematic review of the qualitative evidence to form our own overarching themes relating to the perceptions and experiences of alerts within computerised clinical decision support systems (CCDSS) [20]. We will form these themes using an inductive approach [21] to facilitate the design of future clinical systems to minimise alert fatigue.

Specifically, we will locate and synthesise all qualitative evidence on the use of research evidence on the experience of electronic alerts in primary care. This will include data on

- Barriers and enablers associated with the appropriate use of electronic alerts including aspects of their design and their frequency of appearance.
- The experience of providers in using these alerts to inform their clinical decision-making.

## Methods/design

### Registration

This review will use systematic methodology to synthesise qualitative research evidence and has been registered with the International Prospective Register of Systematic Reviews (PROSPERO, www.crd.york.ac.uk/PROSPERO, CRD42016029418). This protocol is reported in line with PRISMA-P guidelines (Additional file 1), and the review will be reported according to PRISMA guidelines [22].

## Research question: what are the barriers and enablers around the use of alerts/clinical reminders by healthcare professionals?

### Eligibility criteria

Below we outline the criteria against which studies will be included or excluded from the review. In order to be included in the review, the study must meet the following criteria;

#### Types of studies

This review will only include qualitative studies that report primary data. We are defining qualitative studies as those that qualitative methods for data collection and data analysis. This definition has been used in several recent qualitative syntheses [23–25]. Data collection can include but not be limited to semi-structured interviews (regardless of the method of construct analysis) and focus groups. Data analysis can include but not be limited to thematic analysis, grounded theory approach and discourse analysis. We will not include studies where data was collected qualitatively but analysed quantitatively such as via word counts using descriptive statistics. There will be no restriction on the location of the research for study inclusion, and abstracts written in other languages will be translated to assess eligibility. Papers will be included from 1960 (or commencement of database) to capture all initiatives and lessons learnt from alerts during the integration of the electronic health record into the NHS.

#### Types of settings and participants

We will include any qualitative study that consists of the perspective or experience of providers using alerts or reminders either as a standalone study or as part of mixed methods design. Providers are defined as healthcare professionals (to include doctors of all specialities and number of years of training, nurses and allied health professionals). If there is any lack of clarity as to the role of individuals within the study, we will contact the original author to seek clarification.

#### Eligibility criteria

To be eligible for inclusion, all studies must explore the use of electronic alerts or reminders in primary care. This alert or reminder is being used either within a study or as part of routine clinical care. The outcomes are themes on barriers and enablers to adherence to alerts or reminders these can include factors external to the nature of the alert, e.g. characteristics of the provider the primary care environment or patient. Outcomes that will also be included relate to factors inherent to the alert such as its design, the content of text or the frequency with which it is displayed.

### Search strategy

A comprehensive search strategy devised by SB (Information Specialist) and tested by RB (Health Services Researcher) will be used to identify relevant literature. A combination of free-text and index terms will be used in the following databases from 1960 (or commencement of database) to present: MEDLINE (Ovid), MEDLINE In Process (Ovid), EMBASE (Ovid), Health Management Information Consortium (HMIC) (Ovid), PsycINFO (Ovid), Cochrane Library (Wiley) CDSR (Cochrane Database of Systematic Reviews), DARE (Database of Abstracts of Reviews of Effects) and HTA (Health |Technology Assessment Database), Cumulative Index of Nursing and Allied Health (CINAHL) (EBSCO) and Conference Proceedings Citation Index (CPCI) via Web of Science.

The strategy will aim to combine sensitivity of free-text terms, truncation and spelling variations with the precision provided by controlled vocabulary (index terms). Search terms will be tailored to each database, grouped into three categories and combined using Boolean operators. These categories include terms for alerts and reminders, healthcare professionals and attitudes or challenges. Searches in the Ovid platform will where appropriate use the “qualitative (maximises specificity)” filter [26]. A sample search for MEDLINE is included in Additional file 2. There will be a search for grey literature including the OpenGrey database, and the websites of CCDSS suppliers (e.g. EMISHealth (https://www.emishealth.com) and the Phoenix Partnership (SystmOne https://www.tpp-uk.com/products/systmone)). Reference lists of all included articles will be checked for further relevant studies as will studies that cite included articles (using the Science Citation Index via Web of Science).

### Data collection and analysis

In this section, we describe the planned methods for selecting studies and extracting and managing data. We also describe the means by which we will assess the quality of each study included in the review and how we will analyse and present the review findings.

#### Study selection

Search results will be entered into Mendeley Reference Management Software (V1.17.6) where duplicate entries will be removed automatically by an in built algorithm supported by manual checking.

Study selection will be undertaken by two reviewers independently.

1. Titles and abstracts will be screened with reference to the eligibility criteria to remove all ineligible articles. Translation of abstracts will be sought if required, prior to eligibility assessment. The full text of articles remaining will be sought and considered for inclusion in the review using the full eligibility criteria. Part translations of articles not in English will be undertaken to facilitate this process. Disagreements between reviewers will be resolved through discussion and with referral to a third reviewer if required. Articles excluded from the review at the full-text stage will be noted on the data extraction form as will the eligibility criteria these did not satisfy.
2. Analysis will be undertaken using an iterative approach between reviewer one and two to form a pathway of alert usage, with the barriers and enablers marked from the data. Overarching themes from this data will then be established, and this will form the basis of a conceptual framework. Both reviewers will keep the context of the different healthcare systems in mind during this process, and if it is found that some barriers are more applicable to a specific context, for example, healthcare free at the point of access versus insurance based, this will be discussed in the final manuscript.

#### Critical appraisal, quality of reporting and data extraction

The CASP Qualitative Checklist (Critical Appraisal Skills Programme) [27] will be used to assess credibility, transferability, dependability and confirmability within the selected qualitative manuscripts at the study level. Where appropriate, we will also use the Standards for Reporting Qualitative Research [28]. Data from duplicate publications of the same study will be combined rather than choose to assess just one of the publications. Both reviewer one and two will extract data independently using the CASP tool and if necessary, we will approach individual investigators to confirm data and methodology.

Because of the difficulties inherent in assessing every aspect of qualitative, work we will not exclude any based on the quality of their reporting [29] instead we will use a post-synthesis sensitivity analysis as recommended by Carroll et al. [30]. This will allow us to determine whether any of our findings are based on a study of low quality as determined by the review team.

### Data extraction and analysis

Results will be taken from the included papers, including appendices where appropriate, and will be imported into analysis software (NVivo v11). Inductive thematic analysis will then be performed to form overarching third order themes. To enable this, we will use the three stages described by Thomas and Harden [31] in the thematic synthesis of qualitative research. The first is the coding of the findings of the primary studies. The second is the categorisation of these codes into descriptive themes. The third is development of analytical themes to describe the themes that have emerged in the second phase. The approach we adopt will be inductive, i.e. themes emerge from the data through repeated examination and comparison. The emerging descriptive themes will be analysed in consideration of the contextual factors, i.e. healthcare context, setting and reminder usage within clinical decision systems to assess if these contextual factors have any impact upon the ensuing analytical themes. The findings will be verified by utilising independent coding by two reviewers, the triangulation of these codes and iterative discussions amongst all reviewers of the coding framework at each of the three phases of the analysis.

### Presentation of findings and reporting methods

The review will describe participant and setting characteristics, data collection and analysis methods. The findings of the primary papers will be summarised in tabular form describing the key characteristics of each. In addition, we will describe each paper narratively. The review findings will be classified into key themes as informed by the analysis. The review’s findings will also be summarised visually in a proposed conceptual framework explaining the relationship between the key factors influencing the efficacy of electronic alerts.

The protocol was developed and reported according to the Preferred Reporting Items for Systematic Reviews and Meta-Analysis (PRISMA) [22, 32] (Additional file 1). The review methods and results will be reported according to the ENTREQ (enhanced transparency in reporting the synthesis of qualitative research statement) for reporting synthesis of qualitative studies [28]. The final literature searches will be reported using the STARLITE (sampling strategy, type of study, approaches, range of years, limits, inclusions and exclusions, terms used, electronic searches) [33].

## Discussion

Current evidence suggests that alerts have a range of effectiveness [34–38] yet an understanding of the factors that influence discrepancies inefficacy is lacking. There have been several reviews around the use of clinical support tools [34, 35, 39] though there are few high quality reviews which have ultimately attempted to construct a conceptual framework from the qualitative data. Similarly lacking is robust evidence on how the characteristics of alerts and the way they are deployed can impact on the phenomenon of alert fatigue in primary care. Therefore, our review will add considerably to the existing evidence base. The systematic nature of our approach and the comprehensive acquisition of evidence being assessed will provide further insights into how alerts can be altered to improve practitioner and patient outcomes and help explain the heterogeneity in effectiveness and offer suggestions that will increase utility and usability of alerts within CCDSS.

### Research status

At the time of submission, full searches had not been run as per the strategy above and scoping work had been undertaken.

## Additional files

Additional file 1: PRISMA-P reporting schedule. Reporting PRISMA items for protocol using PRISMA P extension. (DOC 83 kb)

Additional file 2: Sample search. Search strategy for MEDLINE. (DOCX 14 kb)

## Abbreviations

CASP: Critical Appraisal Skills Programme
CCDSS: Computerised clinical decision support systems
CINAHL: Cumulative Index to Nursing and Allied Health Literature
CPCI: Conference Proceedings Citation Index
ENTREQ: Enhanced transparency in reporting the synthesis of qualitative research statement
HMIC: Health Management Information Consortium
NHS: National Health Service
NICE: National Institute for Health and Care Excellence
PRISMA: Preferred Reporting Items for Systematic Reviews and Meta-Analyses
PROSPERO: International Prospective Register of Systematic Reviews
RCT: Randomised controlled trial
STARLITE: Sampling strategy, type of study, approaches, range of years, limits, inclusion and exclusions, terms used, electronic sources
UK: United Kingdom
