# Supplementary material for: Clinical reminder alert fatigue in healthcare: a systematic literature review protocol using qualitative evidence
Source: Syst Rev. 2017 Dec 13;6:255. doi: 10.1186/s13643-017-0627-z (PMC5729261; doi:10.1186/s13643-017-0627-z)
Supplement: Supplementary file 2 — Sample search. Search strategy for MEDLINE. (DOCX 14 kb) [file 13643_2017_627_MOESM2_ESM.docx]

Database: MEDLINE (Ovid) 1960/database inception – current.

1. exp Communication Barriers/

2. barrier$.ti,ab.

3. enabler$.ti,ab.

4. facilitator$.ti,ab.

5. attitude$.ti,ab.

6. exp Attitude/

7. exp attitude of health personnel/

8. implementation.ti,ab.

9. physician acceptance.ti,ab.

10. perception$.ti,ab.

11. exp Perception/

12. guideline$.ti,ab.

13. exp Guideline/

14. exp Practice Guideline/

15. exp Guideline Adherence/

16. (lesson$ adj3 learned).ti,ab

17. negative impact.ti,ab.

18. quality improvement.ti,ab.

19. exp Health Services Research/

20. exp Quality Improvement/

21. negative response$.ti,ab.

22. useability.ti,ab.

23. user resistance.ti,ab.

24. challenge$.ti,ab.

25. difficult$.ti,ab.

26. failure$.ti,ab.

27. problem$.ti,ab.

28. OR/1-27

29. alert$.ti,ab.

30. clinical reminder$.ti,ab.

31. exp Reminder Systems/

32. clinical decision support system$.ti,ab.

33. exp Decision Support Systems, Clinical/

34. computerized physician order entry.ti,ab.

35. exp Medical Order Entry Systems/

36. electronic health record$.ti,ab.

37. exp Electronic Health Records/

38. exp Medical Records Systems, Computerized/

39. decision support system$.ti,ab .

40. exp Decision Making, Computer-Assisted/

41. exp Diagnosis, Computer-Assisted/

42. medical record system.ti,ab.

43. exp Information Systems/

44. exp Medical Records/

45. health informatic$.ti,ab.

46. exp Medical Informatics/

47. reminder$.ti,ab.

48. exp Reminders/

49. reminder system$.ti,ab.

50. diagnosis, computer assisted.ti,ab.

51. exp Clinical Alarms/

52. warning$.ti,ab.

53. (alarm adj3 fatigue).ti,ab.

54. algorithm$.ti,ab.

55. exp Algorithms/

56. tool.ti,ab.

57. (alert adj3 fatigue).ti,ab.

58. OR/29-57

59. primary care.ti,ab.

60. exp Primary Health Care/

61. secondary care.ti,ab.

62. exp Secondary Care/

63. (doctor or doctors).ti,ab.]

64. exp Physicians/

65. (nurse or nurses).ti,ab.

66. exp Nurses/

67. healthcare professional$.ti,ab.

68. general practice.ti,ab.

69. general practioner$.ti,ab.

70. exp General Practice/

71. exp Family Practice/

72. (hospital or hospitals).ti,ab.

73. exp Hospitals/

74. medical staff.ti,ab.

75. exp Medical Staff/

76. OR/59-75

77. 28 and 58 and 76

78. limit 77 to yr="1960 - Current"

*(To be run in MEDLINE with additional filter ‘Qualitative (maximises specificity) =)*
